# Bridging the gap: a survey of resident physicians’ needs for cross-sectional anatomy education and a collaborative teaching framework

**DOI:** 10.1186/s12909-026-08567-3

**Published:** 2026-01-07

**Authors:** Zhehua Shao, Jingjie Xu, Jiawei Han, Yu Peng, Xiang Li, Qi Gao, Xuwen Wang, Binben Wang, Duoduo Zhao, Luanqing Che, Chao Zhang

**Affiliations:** 1 Key Laboratory of Respiratory Disease of Zhejiang Province, Department of Respiratory and Critical Care Medicine, Second Affiliated Hospital of Zhejiang University School of Medicine, Hangzhou, Zhejiang China; 2 Eye Center, The Second Affiliated Hospital, School of Medicine, Zhejiang Provincial Key Laboratory of Ophthalmology, Zhejiang Provincial Clinical Research Center for Eye Diseases, Zhejiang University, Zhejiang Provincial Engineering Institute on Eye Diseases, Hangzhou, Zhejiang China; 3 Department of Neurosurgery, Second Affiliated Hospital, School of Medicine, Clinical Research Center for Neurological Disease of Zhejiang Province, Zhejiang University, Hangzhou, China; 4 The Second Affiliated Hospital of Zhejiang University School of Medicine, Hangzhou, Zhejiang China; 5 College of Information Science and Electronic Engineering, Zhejiang University, Hangzhou, China; 6 School of Mathematical Sciences, Zhejiang University, Hangzhou, China; 7 Bone Marrow Transplantation Center of The First Affiliated Hospital, Zhejiang University School of Medicine, Hangzhou, China; 8 Department of Anatomy, Zhejiang University School of Medicine, Hangzhou, Zhejiang China; 9 Department of Pharmacy, Center for Regeneration and Aging Medicine, The Fourth Affiliated Hospital of School of Medicine, and International School of Medicine, International Institutes of Medicine, Zhejiang University, Yiwu, Zhejiang China

**Keywords:** Cross-sectional anatomy, Medical education, Collaborative teaching, Resident training, Imaging education

## Abstract

**Background:**

Cross-sectional anatomy is essential for clinical imaging interpretation, yet many medical curricula lack systematic training for clinical students. This study assessed needs among resident physicians and proposed a collaborative education framework.

**Methods:**

A cross-sectional survey of 130 resident physicians from Zhejiang University-affiliated hospitals (June-August 2025) evaluated knowledge gaps, clinical challenges, and preferences using descriptive statistics, chi-square tests, and logistic regression.

**Results:**

Of 130 respondents (53% female, 58% aged 26–30), 74% reported no formal cross-sectional anatomy training, despite 88% citing high clinical needs. Top challenges included anatomical positioning (45%), with surgery residents showing greatest urgency (95%). Preferences favored clinical-basic science collaboration (64% “very important”), blended online-offline formats (57%), and 3D imaging (71%).

**Conclusions:**

Significant educational gaps persist in cross-sectional anatomy, underscoring the need for collaborative models integrating clinical cases and technology. This framework can guide curriculum reforms to enhance imaging competency and patient safety in global medical education.

**Supplementary Information:**

The online version contains supplementary material available at 10.1186/s12909-026-08567-3.

## Introduction

 Cross-sectional anatomy is a foundational discipline that illustrates the morphological structures and positional relationships of the human body through sectional views [1, 2]. As medical imaging technologies have advanced, cross-sectional anatomy has become an indispensable bridge between traditional anatomical knowledge and radiological interpretation [3]. Proficiency in visualizing and interpreting these structures is now a core competency for healthcare professionals across specialties, enabling accurate image reading and informed clinical decisions [4].

Global utilization of medical imaging has surged, with computed tomography (CT) and magnetic resonance imaging (MRI) volumes increasing 300–400% over the past two decades [5, 6]. This shift has transformed clinical practice, making image interpretation essential not only for radiologists but for all patient-facing physicians. Yet, medical curricula have lagged behind: fewer than 40% of schools worldwide offer systematic cross-sectional anatomy training to all students, often restricting it to radiology residents [7, 8]. This mismatch contributes to diagnostic errors and delays in decision-making, particularly among early-career physicians [9, 10].

Conventional anatomy education inadequately prepares learners for imaging’s spatial demands, as students often memorize isolated structures without grasping three-dimensional relationships [4, 11–13]. Cognitive research confirms spatial visualization requires targeted interventions distinct from rote learning [3, 14, 15].

To address these gaps, international bodies like the LCME and WFME advocate integrating basic and clinical sciences throughout curricula [16, 17]. Collaborative models have proven effective: McMaster University’s radiology-anatomy integration boosted spatial reasoning [14], and Harvard’s approach enhanced learner confidence [18, 19]. Digital tools—3D visualization and virtual reality—further improve spatial skills [20–22], though their impact is maximized when paired with expert guidance and clinical contexts [23, 24].

Despite these advances, learner-centered research on cross-sectional anatomy remains sparse, often prioritizing interventions over needs assessments [25, 26]. Organizations like the Association of American Medical Colleges (AAMC) emphasize curricula grounded in stakeholder input to ensure relevance [27, 28]. Collaborative medical education—engaging clinicians in design and teaching—fosters clinical alignment, boosting engagement and preparation [29, 30]. Successful examples span systems, from UK NHS partnerships to Australian networks, yielding higher satisfaction despite implementation challenges like faculty training [29, 31–34].

Given limited evidence on resident perspectives—the critical transition from education to practice—this study surveyed Zhejiang University-affiliated residents to evaluate knowledge gaps, clinical challenges, and preferences. By focusing on this group, we capture immediate post-graduation insights into curriculum efficacy. Objectives included assessing current training status, identifying needs by specialty and experience, exploring teaching format preferences, and proposing a collaborative framework to integrate anatomical expertise, clinical application, and technology for enhanced imaging competency.

## Materials and methods

### Study design and participants

This cross-sectional survey study was conducted from June to August 2025 at hospitals affiliated with Zhejiang University School of Medicine in Hangzhou, China. The aim was to evaluate knowledge gaps, clinical application needs, and educational preferences for cross-sectional anatomy among resident physicians.

The target population included resident physicians (postgraduate years 1–4 or higher) in training programs at affiliated hospitals. Participants were recruited from diverse clinical departments, including internal medicine, surgery, emergency medicine, radiology, and other specialties, to capture varied experiences with medical imaging interpretation. Inclusion criteria were: (1) active enrollment in a residency program; (2) at least 6 months of clinical experience involving medical imaging; (3) provision of informed consent; and (4) proficiency in Chinese for questionnaire completion. Exclusion criteria included attending physicians or faculty, residents with incomplete training records, and those unable to complete the survey due to scheduling or technical issues.

Sample size calculation was performed using the formula for cross-sectional studies: $$\begin{aligned} \mathrm n=\mathrm Z^2\;\mathrm p\left(1-\mathrm p\right)/\mathrm d^2 \end{aligned}$$, where Z = 1.96 (95% confidence level), *p* = 0.5 (expected proportion, conservative estimate), and d = 0.09 (acceptable margin of error). The calculated minimum sample size was 117 participants. To account for potential non-response rates and incomplete surveys, we aimed to recruit more than 120 participants. Convenience sampling stratified by clinical department ensured representation across specialties.

The study protocol was approved by the Ethics Committee of the Second Affiliated Hospital of Zhejiang University School of Medicine. All participants provided electronic informed consent prior to survey access, and participation was voluntary with the right to withdraw at any time without repercussions.

### Data collection instrument

A structured 24-item questionnaire was developed through literature review and pilot testing with 15 residents. It covered four domains: (1) demographics (5 items: training year, department, age, gender, education level); (2) knowledge and prior education in cross-sectional anatomy (5 items: understanding level, course participation, perceived mastery); (3) clinical application and challenges (7 items: impact, usage frequency, self-assessed proficiency, difficulties); and (4) educational preferences (7 items: content, methods, faculty involvement, formats, technology).

Items used single- or multiple-choice formats, with 5-point Likert scales for attitudes. The instrument was refined based on pilot feedback for clarity and relevance. Psychometric evaluation included content validity (Content Validity Index = 0.94, based on expert ratings), face validity (confirmed via pilot), internal consistency (Cronbach’s α = 0.86), and test-retest reliability (*r* = 0.87 in a subsample of 25 participants retested after 2 weeks). Key items are summarized in Supplementary Table S1.

### Data collection

The questionnaire was administered via Wenjuanxing software (Changsha Ranxing Information Technology Co., Ltd.). Eligible participants were identified by department coordinators and invited via personalized email links containing study details, estimated time (15–20 min), voluntary nature, and support contacts. Surveys were completed anonymously on computers or mobile devices. One- and two-week reminders were sent to non-respondents. The survey ran for 8 weeks, with no incentives offered to reduce selection bias. A total of 130 eligible residents were invited to participate, and all 130 returned fully completed questionnaires (100% response rate, 0 excluded cases). The survey platform required completion of all mandatory items before submission. The complete survey instrument is provided in Additional file 1.

### Statistical analysis

Data were analyzed using GraphPad Prism 9 (GraphPad Software, San Diego, CA, USA). Descriptive statistics summarized categorical variables as frequencies and percentages, and continuous variables as means ± standard deviations. Chi-square tests assessed associations between variables, with Cramér’s V for effect sizes. Logistic regression identified predictors of preferences (e.g., Nagelkerke R² = 0.24 for collaborative teaching). For logistic regression analyses, statistical power was assessed using the events per variable (EPV) approach; with approximately 85 outcome events and 4 predictor variables, the EPV of 21.3 exceeded the recommended minimum of 10, ensuring stable coefficient estimates [35]. Post-hoc power analysis confirmed > 90% power to detect odds ratios ≥ 2.0 at α = 0.05. ANOVA compared group differences in understanding levels. Statistical significance was set at *p* < 0.05 (two-tailed). No adjustments for multiple comparisons were applied due to the exploratory nature.

## Results

### Participant characteristics and cross-sectional anatomy educational background

Of 130 resident physicians who completed the survey (100% response rate), the sample showed balanced demographics with slight female predominance (53.1%, *n* = 69) and most aged 26–30 years (58.5%, *n* = 76). Training year distribution was first-year (30.0%, *n* = 39), second-year (31.5%, *n* = 41), third-year (23.1%, *n* = 30), and fourth-year or above (15.4%, *n* = 20). Departments included other specialties (35.4%, *n* = 46), internal medicine (33.8%, *n* = 44), and surgery (30.8%, *n* = 40). Educational levels comprised doctoral degrees (45.4%, *n* = 59), bachelor’s (34.6%, *n* = 45), and master’s (20.0%, *n* = 26) (Table 1).

**Table 1:** Participant characteristics and Cross-sectional anatomy background (*N* = 130)

Characteristic	*n*	%
Gender		
Male	61	46.92
Female	69	53.08
Age (years)		
20–25	25	19.23
26–30	79	58.46
31–35	27	20.77
36 and above	2	1.54
Training Year		
1st year	39	30
2nd year	41	31.54
3rd year	30	23.08
4th year and above	20	15.38
Department		
Internal Medicine	44	33.85
Surgery	40	30.77
Other specialties	46	35.38

**Fig. 1:**
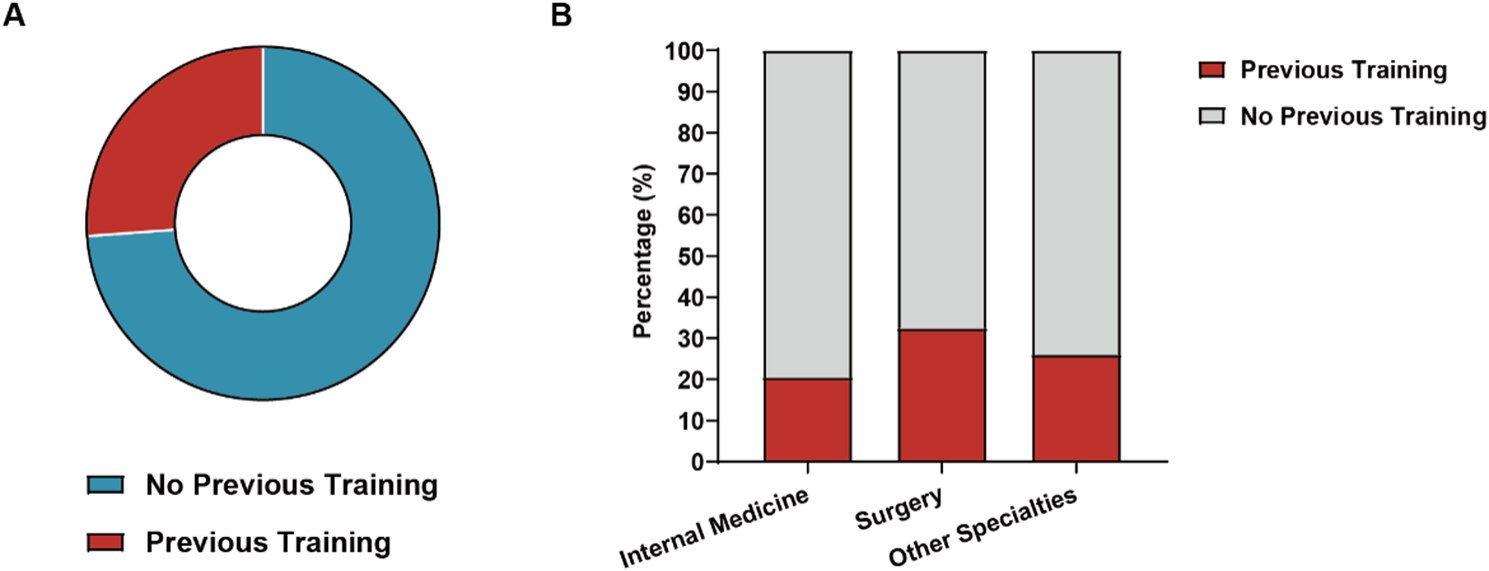
Educational Background and Departmental Variations. **A** Prior training exposure and self-reported knowledge distribution (bar chart). **B** Departmental participation rates (stacked bar), highlighting surgery’s higher exposure (32.5%)

Formal cross-sectional anatomy training was limited, with 73.8% (*n* = 96) reporting none and only 26.2% (*n* = 34) having systematic exposure (Fig. 1A). Self-reported understanding (4-point scale) indicated gaps: 53.9% (*n* = 70) “somewhat knowledgeable”, 36.2% (*n* = 47) “not very knowledgeable”, 5.4% (*n* = 7) “not at all”, and 4.6% (*n* = 6) “very knowledgeable”. Departmental differences in prior participation were significant (χ²=8.94, *p* = 0.011), with internal medicine residents highest (31.8%), but understanding levels did not vary substantially (F = 2.14, *p* = 0.098) (Fig. 1B, and Table 2).

**Table 2:** Cross-sectional anatomy educational background by department (*N* = 130)

Previous Course Participation							
	Internal Medicine	Surgery	Other Specialties	Total	χ² (df)	*p*-value	Cramér’s V
	*n* = 44	*n* = 40	*n* = 46	*N* = 130			
Yes	14 (31.8%)	11 (27.5%)	9 (19.6%)	34 (26.1%)	8.94 (2)	0.011*	0.26 (Small)
No	30 (68.2%)	29 (72.5%)	37 (80.4%)	96 (73.8%)			

Self-reported Understanding Level							
	Internal Medicine	Surgery	Other Specialties	Total	χ² (df)	F (df)	Effect
	*n* = 44	*n* = 40	*n* = 46	*N* = 130			
Very knowledgeable	1 (2.3%)	2 (5.0%)	3 (6.5%)	6 (4.6%)	2.14 (2, 127)	0.098	n.s.
Somewhat knowledgeable	25 (56.8%)	24 (60.0%)	21 (45.6%)	70 (53.9%)			
Not very knowledgeable	15 (34.1%)	12 (30.0%)	20 (43.5%)	47 (36.1%)			
Not knowledgeable at all	3 (6.8%)	2 (5.0%)	2 (4.3%)	7 (5.4%)			

Percentages are calculated within each department (column percentages)

Effect size for Cramér’s V (Cohen, 1988): <0.10 = negligible, 0.10–0.29 = small, 0.30–0.49 = medium, ≥ 0.50 = large

Internal Medicine residents showed the highest rate of prior course participation (31.8%), significantly different across departments

*p < 0.05; n.s. = not significant

### Clinical application challenges and training progression

Anatomical structure positioning and adjacent relationships represented the dominant challenge across all groups (45.38%, *n* = 59), with distinct departmental patterns. Surgery residents demonstrated highest rates of anatomical positioning challenges (52.5%, *n* = 21), while internal medicine residents emphasized pathological changes assessment (40.9%, *n* = 18), and other specialties focused on comprehensive diagnosis difficulties (43.5%, *n* = 20) (χ² = 11.2, *p* = 0.024).

An overwhelming 88.46% (*n* = 115) reported high or very high educational needs, with surgery residents showing the greatest urgency (95.0%, *n* = 38), significantly exceeding internal medicine (86.4%, *n* = 38) and other specialties (84.8%, *n* = 39) (χ² = 12.3, *p* = 0.006). This need intensity correlated strongly with clinical exposure frequency and perceived knowledge gaps.

Analysis of questionnaire responses revealed actual understanding progression patterns: First-year residents (*n* = 39): Mean = 2.67 ± 0.69 (4-point scale); Second-year residents (*n* = 41): Mean = 2.59 ± 0.62; Third-year residents (*n* = 30): Mean = 2.73 ± 0.63; Fourth-year and above (*n* = 20): Mean = 2.65 ± 0.65. Contrary to expected linear progression, ANOVA revealed no significant difference in understanding levels across training years (F = 0.34, *p* = 0.718), indicating persistent knowledge gaps throughout residency training (Fig. 1A, and Table 3).

**Table 3:** Clinical competency indicators by training year (*N* = 130)

Variable	1st Year (*n* = 39)	2nd Year (*n* = 41)	3rd Year (*n* = 30)	4th + Year (*n* = 20)	Statistic (df)	*p*-value	Effect Size
Understanding Level (4-point scale)							
Mean ± SD	2.67 ± 0.69	2.59 ± 0.62	2.73 ± 0.63	2.65 ± 0.65	F = 0.34 (3, 126)	0.718	*η*² = 0.008 (Negligible)

Independent Reading Confidence							
Confident, n (%)	9 (23.1)	12 (29.3)	12 (40.0)	11 (55.0)	χ² = 7.43 (3)	0.041*	V = 0.24 (Small)
Primary Clinical Challenges, n (%)							
Anatomical Positioning	18 (46.2)	19 (46.3)	13 (43.3)	9 (45.0)	χ² = 0.12 (3)	0.989	V = 0.03 (Negligible)
Pathological Assessment	9 (23.1)	9 (22.0)	7 (23.3)	5 (25.0)	χ² = 0.09 (3)	0.993	V = 0.03 (Negligible)
Comprehensive Diagnosis	9 (23.1)	10 (24.4)	7 (23.3)	5 (25.0)	χ² = 0.03 (3)	0.999	V = 0.02 (Negligible)
Lesion Localization	3 (7.7)	3 (7.3)	3 (10.0)	1 (5.0)	χ² = 0.35 (3)	0.950	V = 0.05 (Negligible)

Understanding level measured on 4-point scale: 1 = not knowledgeable at all, 2 = not very knowledgeable, 3 = somewhat knowledgeable, 4 = very knowledgeable

Effect size interpretation: Cramér’s V: <0.10 = negligible, 0.10–0.29 = small, 0.30–0.49 = medium, ≥ 0.50 = large (Cohen, 1988)

*η*² (eta-squared): <0.01 = negligible, 0.01–0.06 = small, 0.06–0.14 = medium, ≥ 0.14 = large

*df* degrees of freedom, *SD* standard deviation

*p < 0.05

Confidence in independent imaging interpretation showed clear progressive improvement: First-year: 9/39 residents (23.1%) confident; Second-year: 12/41 residents (29.3%) confident; Third-year: 12/30 residents (40.0%) confident; Fourth-year and above: 11/20 residents (55.0%) confident. Chi-square analysis confirmed significant training year effects on confidence (χ² = 7.43, *p* = 0.041), with confidence more than doubling from first to fourth year (Fig. 1B, and Table 3).

**Fig. 2:**
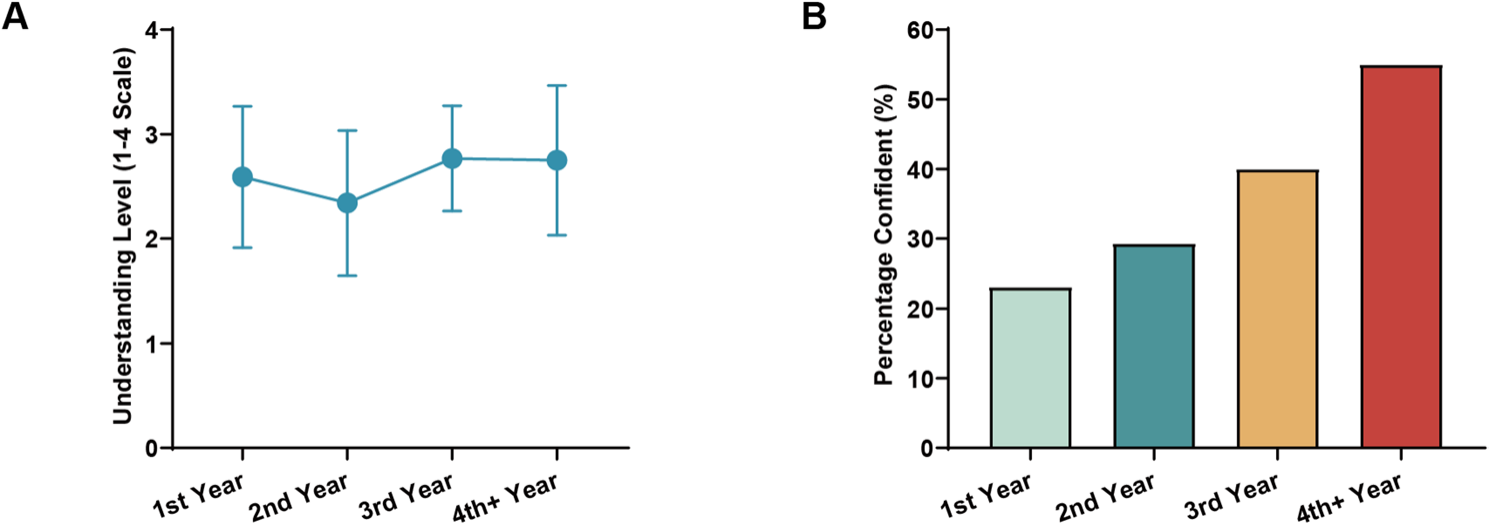
Competency Development Across Training Years. **A **Stable understanding scores (means ± SD; 4-point scale). **B** Increasing independent reading confidence (% confident)

Residents with prior cross-sectional anatomy training (*n* = 34) demonstrated superior outcomes compared to those without (*n* = 96): higher self-assessed reading proficiency (41.2% vs. 21.9% “quite proficient” or above, *p* = 0.008) and significantly greater confidence in independent reading (47.1% vs. 28.1%, *p* = 0.003) (Fig. 2).

### Educational preferences and collaborative teaching support analysis

**Fig. 3:**
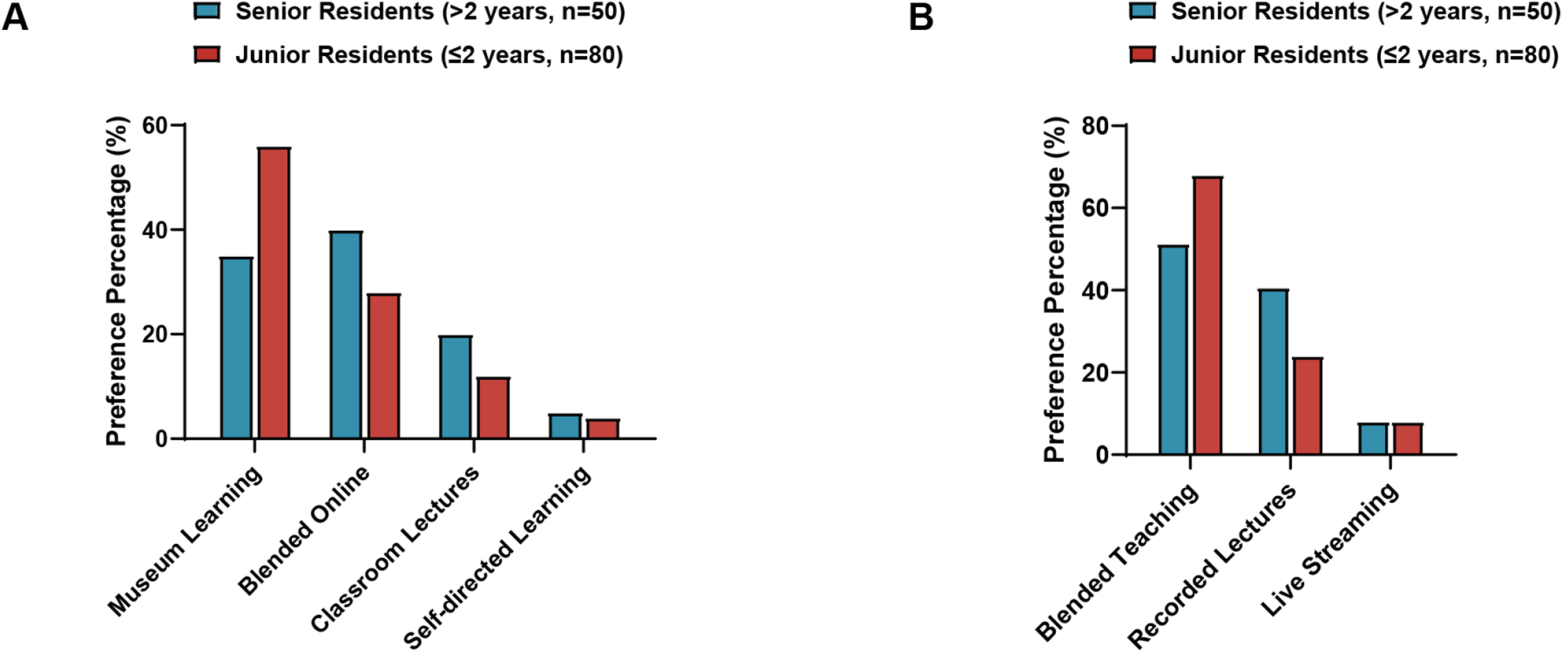
Educational format preferences by experience level. **A** Learning format preferences among all participants (*N* = 130). Senior residents showed significantly higher preference for museum-based learning (56.0% vs. 35.0%, *p* = 0.027). **B** Online course format preferences among residents selecting online modalities (*n* = 74). Senior residents demonstrated stronger preference for blended teaching approaches (68.0% vs. 51.3%, *p* = 0.025). *p < 0.05

Human anatomy museum-based learning emerged as the preferred format (43.08%, *n* = 56), followed by blended online-offline approaches (35.38%, *n* = 46), classroom lectures (16.92%, *n* = 22), and self-directed learning (4.62%, *n* = 6). Senior residents (3rd + year) showed significantly higher preference for museum-based learning (56.0% vs. 35.0%, χ² = 4.86, *p* = 0.027) (Fig. 3A, and Table 4).

**Table 4:** Educational preferences and collaborative teaching support by demographics (*N* = 130)

Learning Format Preferences							
Group	Museum Learning	Blended Online	Classroom	Self- directed	χ² (df)	*p*-value	Effect Size
All Participants (*N* = 130)	56 (43.1%)	46 (35.4%)	22 (16.9%)	6 (4.6%)			
By Experience Level							
Junior (≤ 2 years, *n* = 80)	28 (35.0%)	32 (40.0%)	16 (20.0%)	4 (5.0%)	4.86 (1)	0.027*	V = 0.19 (Small)
Senior (> 2 years, *n* = 50)	28 (56.0%)*	14 (28.0%)	6 (12.0%)	2 (4.0%)			
By Gender							
Male (*n* = 61)	17 (27.9%)	21 (34.4%)*	15 (24.6%)	8 (13.1%)	See note†		
Female (*n* = 69)	27 (39.1%)*	17 (24.6%)	13 (18.8%)	12 (17.4%)			

Importance of Clinical Physician Participation							
Group	Very Important	Important	Average	Less/Not Important	χ² (df)	*p*-value	Effect Size
All Participants (*N* = 130)	83 (63.8%)	41 (31.5%)	6 (4.6%)	0 (0%)			
By Experience Level							
Junior (≤ 2 years, *n* = 80)	52 (65.0%)	25 (31.3%)	3 (3.8%)	0 (0%)	0.03 (1)	0.874	n.s.
Senior (> 2 years, *n* = 50)	31 (62.0%)	16 (32.0%)	3 (6.0%)	0 (0%)			
By Department							
Internal Medicine (*n* = 44)	27 (61.4%)	15 (34.1%)	2 (4.5%)	0 (0%)	0.18 (2)	0.915	n.s.
Surgery (*n* = 40)	26 (65.0%)	13 (32.5%)	1 (2.5%)	0 (0%)			
Other Specialties (*n* = 46)	30 (65.2%)	13 (28.3%)	3 (6.5%)	0 (0%)			

Support for Collaborative Clinical-Anatomy Teaching							
Group	Strongly Support	Support	Average	Strongly Oppose	χ² (df)	*p*-value	Effect Size
All Participants (*N* = 130)	85 (65.4%)	40 (30.8%)	5 (3.8%)	0 (0%)			
By Experience Level							
Junior (≤ 2 years, *n* = 80)	52 (65.0%)	25 (31.3%)	3 (3.8%)	0 (0%)	0.00 (1)	1.000	n.s.
Senior (> 2 years, *n* = 50)	33 (66.0%)	15 (30.0%)	2 (4.0%)	0 (0%)			
By Department							
Internal Medicine (*n* = 44)	27 (61.4%)	15 (34.1%)	2 (4.5%)	0 (0%)	0.69 (2)	0.708	n.s.
Surgery (*n* = 40)	28 (70.0%)	11 (27.5%)	1 (2.5%)	0 (0%)			
Other Specialties (*n* = 46)	30 (65.2%)	14 (30.4%)	2 (4.3%)	0 (0%)			

Effect size interpretation for Cramér’s V (Cohen, 1988): <0.10 = negligible, 0.10–0.29 = small, 0.30–0.49 = medium, ≥ 0.50 = large

No significant differences were found in collaborative teaching support across experience levels or departments

*p < 0.05; n.s. = not significant

†Gender differences in learning preferences: Museum learning (*p* = 0.043), Blended online (*p* = 0.036)

Notable gender differences emerged in delivery method preferences rather than content requirements. Females demonstrated significantly higher preference for anatomy museum learning (39.1% vs. 27.9%, *p* = 0.043), while males favored online courses (34.4% vs. 24.6%, *p* = 0.036). These differences suggest the importance of offering diverse learning modalities (Table 4).

Among participants selecting online modalities (*n* = 74), blended teaching dominated preferences (56.8%, *n* = 42), followed by recorded lectures (33.8%, *n* = 25) and live streaming (9.5%, *n* = 7). Senior residents demonstrated even stronger preference for blended approaches (68.0% vs. 51.3%, *p* = 0.025), suggesting greater appreciation for integrated learning methods (Fig. 3B).

Strong endorsement emerged for collaborative approaches, with 63.85% (*n* = 83) rating clinical physician participation as “very important” and 65.38% (*n* = 85) providing “strong support” for collaborative clinical-anatomy teaching. Support intensity was consistent across experience levels, with junior residents (65.0%, *n* = 52) and senior residents (62.0%, *n* = 31) showing similar “very important” ratings (χ² = 0.03, *p* = 0.874). No significant departmental differences were observed (*p* = 0.915) (Table 4).

### Course content requirements and technology integration demands

Strong consensus emerged for clinically relevant content integration: clinical case imaging anatomy analysis received overwhelming support (90.77%, *n* = 118), multi-modal imaging anatomy comparison (80.00%, *n* = 104), and imaging anatomy-surgical correlation (70.77%, *n* = 92). These preferences remained consistent across all departments and training levels.

Three-dimensional imaging technology application was desired by 70.77% (*n* = 92), with senior residents showing significantly higher preference (84.0% vs. 64.1%, *p* = 0.005). Interactive teaching methods were favored by 52.31% (*n* = 68), with males demonstrating significantly greater interest than females (59.0% vs. 44.9%, *p* = 0.031). Doctoral degree holders showed enhanced preference for advanced technology integration (79.7% vs. 62.0%, *p* = 0.015).

Perceived advantages of collaborative teaching showed remarkable consistency: providing more clinical cases received highest recognition (90.77%, *n* = 118), followed by better integration with practical operations (86.15%, *n* = 112) and provision of multi-perspective knowledge (74.62%, *n* = 97). Surgery residents demonstrated highest appreciation across all collaborative teaching dimensions (Table 5).

**Table 5:** Technology integration preferences and content requirements by demographics (*N* = 130)

Demographic Variable	Core Technology Preferences (%)			Advanced Technology Interest (%)		
	3D Imaging	Interactive Teaching	VR Applications	AR Visualization	AI- assisted	Virtual Surgery
Overall Distribution						
All Participants (*N* = 130)	92 (70.8%)	68 (52.3%)	45 (34.6%)	45 (34.6%)	38 (29.2%)	33 (25.4%)
Experience Level Analysis						
Junior (≤ 2 years, *n* = 80)	51 (64.1%)	37 (46.3%)	26 (32.5%)	24 (30.0%)	21 (26.3%)	17 (21.3%)
Senior (> 2 years, *n* = 50)	42 (84.0%)*	31 (62.0%)	19 (38.0%)	21 (42.0%)	17 (34.0%)	16 (32.0%)
Statistical Test	χ²=5.89 (1), *p* = 0.005	n.s.	n.s.	n.s.	n.s.	n.s.
Effect Size	V = 0.21 (Small)	—	—	—	—	—
Gender Differences						
Male (*n* = 61)	47 (77.0%)	36 (59.0%)*	25 (41.0%)	24 (39.3%)	20 (32.8%)	18 (29.5%)
Female (*n* = 69)	45 (64.9%)	31 (44.9%)	20 (29.0%)	21 (30.4%)	18 (26.1%)	15 (21.7%)
Statistical Test	n.s.	χ²=4.65 (1), *p* = 0.031	n.s.	n.s.	n.s.	n.s.
Effect Size	—	V = 0.19 (Small)	—	—	—	—
Educational Level						
Doctoral (*n* = 59)	47 (79.7%)*	35 (59.3%)	23 (39.0%)	24 (40.7%)	21 (35.6%)	18 (30.5%)
Non-doctoral (*n* = 71)	44 (62.0%)	33 (46.5%)	22 (31.0%)	21 (29.6%)	17 (23.9%)	15 (21.1%)
Statistical Test	χ²=5.92 (1), *p* = 0.015	n.s.	n.s.	n.s.	n.s.	n.s.
Effect Size	V = 0.21 (Small)	—	—	—	—	—
Department Distribution						
Internal Medicine (*n* = 44)	30 (68.2%)	21 (47.7%)	14 (31.8%)	14 (31.8%)	11 (25.0%)	10 (22.7%)
Surgery (*n* = 40)	30 (75.0%)	23 (57.5%)	16 (40.0%)	16 (40.0%)	13 (32.5%)	12 (30.0%)
Other Specialties (*n* = 46)	32 (69.6%)	24 (52.2%)	15 (32.6%)	15 (32.6%)	14 (30.4%)	11 (23.9%)
Statistical Test	n.s.	n.s.	n.s.	n.s.	n.s.	n.s.

Effect size interpretation for Cramér’s V (Cohen, 1988): <0.10 = negligible, 0.10–0.29 = small, 0.30–0.49 = medium, ≥ 0.50 = large

Percentages may not sum to 100% as participants could select multiple options

*VR* Virtual Reality, *AR* Augmented Reality, *AI* Artificial Intelligence

*p < 0.05; n.s. = not significant; — = not applicable

Beyond basic technology integration, participants expressed interest in cutting-edge educational tools: augmented reality visualization (34.62%, *n* = 45), artificial intelligence-assisted diagnosis training (29.23%, *n* = 38), and virtual surgical planning integration (25.38%, *n* = 33) [35]. These preferences correlated positively with educational level (*r* = 0.31, *p* < 0.001) and training progression (*r* = 0.29, *p* = 0.001).

The survey instrument demonstrated excellent psychometric properties (Cronbach’s α = 0.86). Key statistical associations included significant association between previous training and confidence levels (*r* = 0.38, *p* < 0.001), and correlation between training year and technology preferences (*r* = 0.29, *p* = 0.001). Notably, collaborative teaching support remained consistently high across all experience levels and departments (> 60% rating “very important”), indicating universal endorsement regardless of training stage (χ² = 0.03, *p* = 0.874). The most consistent findings across all analyses showed that surgery residents demonstrated highest needs and support across all measured dimensions, senior residents exhibited stronger preferences for specific learning modalities (museum-based learning: 56.0% vs. 35.0%, *p* = 0.027; 3D imaging: 84.0% vs. 64.1%, *p* = 0.005), previous training experience produced significant positive effects on competency measures, and doctoral degree holders showed stronger support for advanced educational approaches.

## Discussion

This study confirms substantial gaps in cross-sectional anatomy education, with 74% of resident physicians lacking formal training despite 88% reporting high clinical needs. These findings echo global reports of curricular shortcomings. European studies have documented similar challenges: the German Radiological Society’s curriculum framework emphasizes integrating cross-sectional anatomy with clinical radiology training [36], while Scandinavian programs advocate early imaging education for medical students [37]. A Polish study further highlighted the need for innovative, multimodal approaches to anatomy instruction [38]. These international parallels underscore patient safety risks, especially in imaging-dependent specialties where spatial reasoning errors can delay diagnoses [7–10, 39].

The pronounced educational needs among surgical residents (95.0%) likely reflect the demands of operative practice, where precise anatomical localization is essential for incision planning and intraoperative navigation, particularly with the increasing adoption of minimally invasive techniques requiring three-dimensional spatial reasoning from two-dimensional images. Several contextual factors may distinguish China’s setting: traditional curricula emphasizing cadaveric dissection with limited cross-sectional imaging integration, high patient volumes constraining self-directed learning time, and rapid adoption of advanced imaging technologies outpacing curriculum updates. These factors suggest that while our findings align with global trends, implementation strategies may require adaptation to local healthcare structures.

The proposed collaborative framework bridges basic anatomy and clinical application by integrating faculty from both domains. Participants universally endorsed its benefits across all experience levels and departments (> 60% rating clinical physician participation as “very important,” with no significant differences between junior and senior residents, *p* = 0.874)—enhanced clinical case integration, practical workflow alignment, and multi-perspective insights—aligning with constructivist principles emphasizing active knowledge construction through authentic contexts [32].

**Fig. 4:**
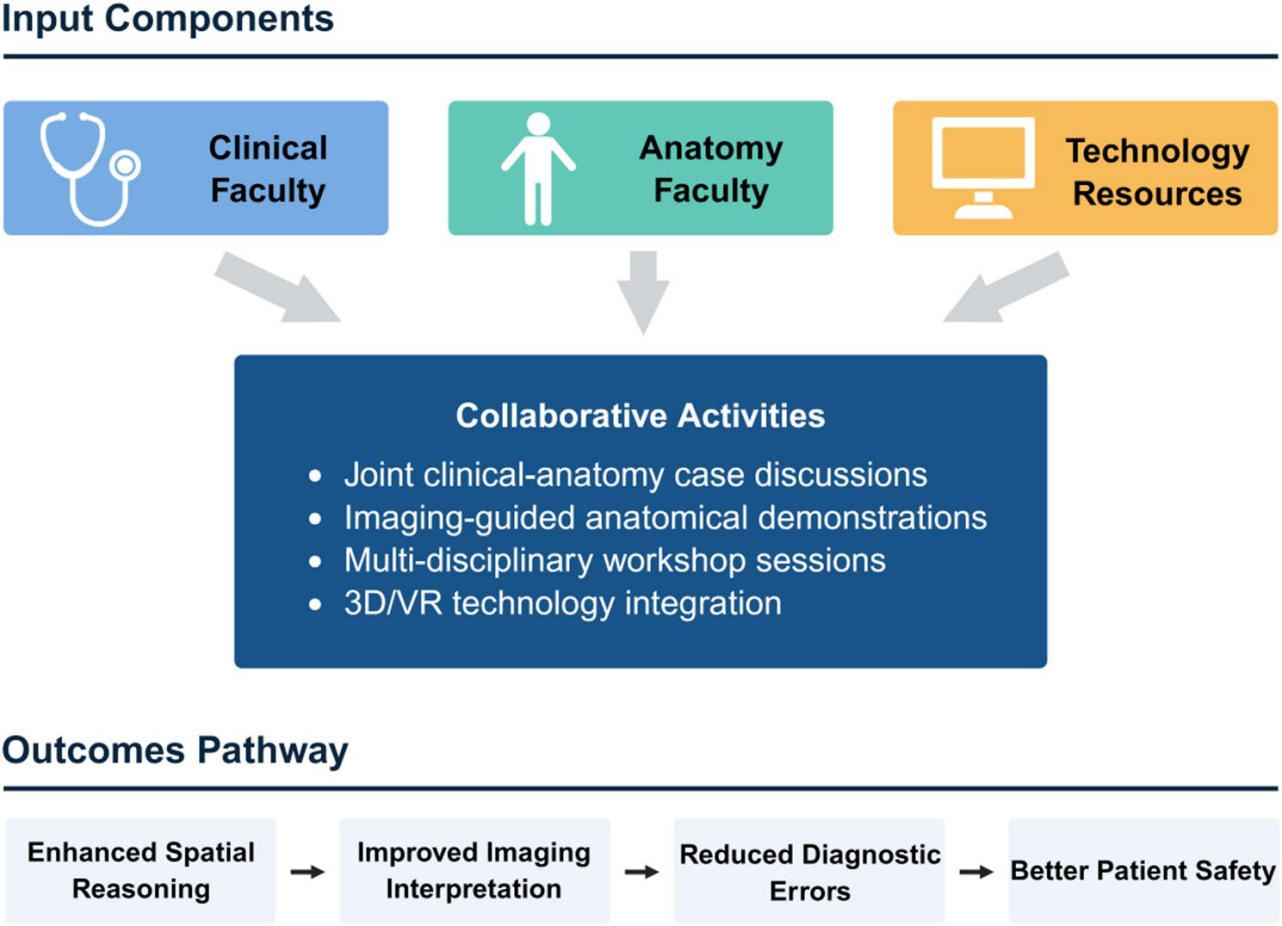
Schematic diagram of the proposed collaborative cross-sectional anatomy education framework. The framework integrates clinical faculty, anatomy faculty, and technology resources through collaborative activities including case discussions, imaging-guided demonstrations, and multi-disciplinary workshops, aiming to enhance spatial reasoning, improve imaging interpretation, and ultimately improve patient safety

Specialty-specific patterns advocate for tailored modules rather than uniform curricula, while strong preferences for 3D imaging and interactive methods signal readiness for digital upgrades. Gender variations in format choices suggest inclusive designs to optimize engagement. Collectively, these insights endorse pre-practice competency assessments in cross-sectional anatomy, harmonizing with competency-based education mandates [16, 27]. The framework envisions a blended structure: 60% case-based clinical-anatomy sessions, 30% technology-enhanced modules (e.g., 3D/VR simulations), and 10% formative evaluations, scalable for resource-limited settings (Fig. 4).

This collaborative framework is designed to supplement, rather than replace, existing anatomy and radiology curricula. The modular structure allows flexible integration into current residency programs through departmental teaching rounds, self-paced online learning, and periodic hands-on workshops, minimizing disruption to clinical duties while addressing the specific gap in cross-sectional imaging interpretation that emerges during clinical practice.

### Limitations

This single-institution study may limit generalizability, though the robust sample (*N* = 130) and stratified demographics enhance internal validity. Self-reported data risk response bias, and the cross-sectional design precludes causal or longitudinal inferences. Selection bias may exist, as residents with greater interest in imaging education might have been more motivated to participate, potentially leading to over-reporting of educational needs. Additionally, our reliance on self-reported measures without objective knowledge assessments (e.g., standardized anatomy tests or image interpretation evaluations) limits the ability to correlate perceived needs with actual competency gaps. The online survey methodology, while enabling broad reach and anonymity, may introduce coverage bias and lacks the depth achievable through qualitative interviews. Future research should include: (1) detailed analysis of educational needs across individual specialties beyond the broad categories examined here, enabling more precisely tailored curricula; (2) pilot implementation studies with pre- and post-training evaluations to assess framework effectiveness; and (3) multicenter or international validation to enhance generalizability across different healthcare systems and cultural contexts. Our results align with WFME standards for integrated curricula, implying broader applicability beyond China to address global imaging education disparities. By prioritizing learner needs, this work advances evidence-based reforms to prepare physicians for technology-driven practice [5, 6, 16].

## Conclusion

In conclusion, this study highlights urgent gaps in cross-sectional anatomy education among resident physicians and endorses a collaborative, technology-enhanced framework to address clinical demands. By integrating clinical cases, multi-disciplinary faculty, and digital tools like 3D imaging, such reforms can enhance spatial reasoning, imaging proficiency, and patient safety. These findings provide actionable guidance for curriculum developers worldwide, advancing competency-based medical education in an era of escalating imaging reliance.

## Supplementary Information

Supplementary Material 1.

## Data Availability

The datasets generated and analysed during the current study are not publicly available due to privacy considerations but are available from the corresponding author on reasonable request.

## References

[1] Ogut E, Yildirim FB, Senol Y, Senol AU. Comprehensive evaluation of the educational impact and effectiveness of specialized study modules in cross-sectional anatomy: a study on student engagement and learning outcomes. BMC Med Educ. 2025;25(1):514. 40211255 10.1186/s12909-025-07050-9 PMC11987277

[2] Samarakoon LB, Vithoosan S, Kokulan S, Dissanayake MM, Anthony DJ, Dissanayake V, Jayasekara R. Anatomy of Teaching Anatomy: Do Prosected Cross Sections Improve Students Understanding of Spatial and Radiological Anatomy? Anatomy research international. 2016;2016:8984704. 10.1155/2016/8984704 PMC4992790 27579181

[3] Murphy KP, Crush L, O’Malley E, Daly FE, O’Tuathaigh CM, O’Connor OJ, Cryan JF, Maher MM. Medical student knowledge regarding radiology before and after a radiological anatomy module: implications for vertical integration and self-directed learning. Insights into Imaging. 2014;5(5):629–34. 25107581 10.1007/s13244-014-0346-0 PMC4195841

[4] Miller SA, Perrotti W, Silverthorn DU, Dalley AF, Rarey KE. From college to clinic: reasoning over memorization is key for Understanding anatomy. Anat Rec. 2002;269(2):69–80. 12001213 10.1002/ar.10071

[5] Smith-Bindman R, Kwan ML, Marlow EC, Theis MK, Bolch W, Cheng SY, Bowles EJA, Duncan JR, Greenlee RT, Kushi LH, et al. Trends in use of medical imaging in US health care systems and in Ontario, Canada, 2000–2016. JAMA. 2019;322(9):843–56. 31479136 10.1001/jama.2019.11456 PMC6724186

[6] Smith-Bindman R, Miglioretti DL, Larson EB. Rising use of diagnostic medical imaging in a large integrated health system. Health Aff. 2008;27(6):1491–502. 10.1377/hlthaff.27.6.1491 PMC2765780 18997204

[7] Shin M, Prasad A, Sabo G, Macnow ASR, Sheth NP, Cross MB, Premkumar A. Anatomy education in US medical schools: before, during, and beyond COVID-19. BMC Med Educ. 2022;22(1):103. 35172819 10.1186/s12909-022-03177-1 PMC8851737

[8] McBride JM, Drake RL. National survey on anatomical sciences in medical education. Anat Sci Educ. 2018;11(1):7–14. 29265741 10.1002/ase.1760

[9] Turney BW. Anatomy in a modern medical curriculum. Ann R Coll Surg Engl. 2007;89(2):104–7. 17346399 10.1308/003588407X168244 PMC1964553

[10] Koppes DM, Triepels CPR, Notten KJB, Smeets CFA, Kruitwagen R, Van Gorp T, Scheele F, Van Kuijk SMJ. The level of anatomical Knowledge, hard to establish: a systematic narrative review. Med Sci Educ. 2022;32(2):569–81. 35528299 10.1007/s40670-022-01509-w PMC9054958

[11] Zhang M, Yu Y, Sun B, Xiao C, Yang J, Yu Z, Yang D. Investigating Clinical-Relevant learning in the anatomy curriculum: perspectives and effectiveness for undergraduate medical students. J Med Educ Curric Dev. 2025;12:23821205251328952. 40124117 10.1177/23821205251328952 PMC11926839

[12] Gonzales RA, Ferns G, Vorstenbosch M, Smith CF. Does Spatial awareness training affect anatomy learning in medical students? Anat Sci Educ. 2020;13(6):707–20. 32048478 10.1002/ase.1949

[13] Roach VA, Fraser GM, Kryklywy JH, Mitchell DG, Wilson TD. The eye of the beholder: can patterns in eye movement reveal aptitudes for Spatial reasoning? Anat Sci Educ. 2016;9(4):357–66. 26599398 10.1002/ase.1583

[14] Roach VA, Mi M, Mussell J, Van Nuland SE, Lufler RS, DeVeau KM, Dunham SM, Husmann P, Herriott HL, Edwards DN, et al. Correlating Spatial ability with anatomy assessment performance: A Meta-Analysis. Anat Sci Educ. 2021;14(3):317–29. 33124194 10.1002/ase.2029 PMC9039732

[15] Koh MY, Tan GJS, Mogali SR. Spatial ability and 3D model colour-coding affect anatomy performance: a cross-sectional and randomized trial. Sci Rep. 2023;13(1):7879. 37188811 10.1038/s41598-023-35046-2 PMC10185657

[16] Bórquez RL. The WFME basic medical education standards on the horizon 2030. Med Sci Educ. 2023;33(Suppl 1):15–8. 10.1007/s40670-023-01947-0 PMC10858850 38347870

[17] Functions and Structure of a Medical School. Standards for Accreditation of Medical Education Programs Leading to the MD Degree. https://lcme.org/publications/.

[18] McDaniel KG, Brown T, Radford CC, McDermott CH, van Houten T, Katz ME, Stearns DA, Hildebrandt S. Anatomy as a model environment for acquiring professional competencies in medicine: experiences at Harvard medical school. Anat Sci Educ. 2021;14(2):241–51. 32657538 10.1002/ase.2000

[19] Melbourne Uo. Master of medicine (Radiology) course handbook. Melbourne: University of Melbourne; 2024.

[20] Wang J, Li W, Dun A, Zhong N, Ye Z. 3D visualization technology for learning human anatomy among medical students and residents: a meta- and regression analysis. BMC Med Educ. 2024;24(1):461. 38671399 10.1186/s12909-024-05403-4 PMC11055294

[21] Lewis TL, Burnett B, Tunstall RG, Abrahams PH. Complementing anatomy education using three-dimensional anatomy mobile software applications on tablet computers. Clin Anat (New York NY). 2014;27(3):313–20. 10.1002/ca.22256 23661327

[22] Yammine K, Violato C. A meta-analysis of the educational effectiveness of three-dimensional visualization technologies in teaching anatomy. Anat Sci Educ. 2015;8(6):525–38. 25557582 10.1002/ase.1510

[23] Grainger R, Liu Q, Gladman T. Learning technology in health professions education: realising an (un)imagined future. Med Educ. 2024;58(1):36–46. 37555302 10.1111/medu.15185

[24] Guze PA. Using technology to Meet the challenges of medical education. Trans Am Clin Climatol Assoc. 2015;126:260–70. 26330687 PMC4530721

[25] Gupta S, Gupta AK, Verma M, Kaur H, Kaur A, Singh K. The attitudes and perceptions of medical students towards basic science subjects during their clinical years: A cross-sectional survey. Int J Appl Basic Med Res. 2014;4(1):16–9. 24600572 10.4103/2229-516X.125675 PMC3931207

[26] Evans DJR, Bay BH, Wilson TD, Smith CF, Lachman N, Pawlina W. Going virtual to support anatomy education: A STOPGAP in the midst of the Covid-19 pandemic. Anat Sci Educ. 2020;13(3):279–83. 32277598 10.1002/ase.1963

[27] The Core Competencies and What They Mean. https://case.edu/studentlife/educationabroad/sites/default/files/2023-08/AAMC%20Core%20Competencies%20%281%29.pdf.

[28] Raffing R, Larsen S, Konge L, Tønnesen H: From targeted needs assessment to course ready for implementation-a model for curriculum development and the course results. Int J Environ Res Public Health. 2023;20(3):2529. 10.3390/ijerph20032529 PMC9915190 36767895

[29] Chandrashekar A, Mohan J. Preparing for the National health service: the importance of teamwork training in the united Kingdom medical school curriculum. Adv Med Educ Pract. 2019;10:679–88. 31686942 10.2147/AMEP.S203333 PMC6709809

[30] Steinert Y, Mann K, Anderson B, Barnett BM, Centeno A, Naismith L, Prideaux D, Spencer J, Tullo E, Viggiano T, et al. A systematic review of faculty development initiatives designed to enhance teaching effectiveness: A 10-year update: BEME guide 40. Med Teach. 2016;38(8):769–86. 27420193 10.1080/0142159X.2016.1181851

[31] Denton GD, Seoane L, Eley DS. Ten years of a Trans-Pacific medical education Partnership-Training globally to serve locally. Ochsner J. 2023;23(4):296–303. 38143540 10.31486/toj.23.0081 PMC10741811

[32] Oudbier J, Verheijck E, van Diermen D, Tams J, Bramer J, Spaai G. Enhancing the effectiveness of interprofessional education in health science education: a state-of-the-art review. BMC Med Educ. 2024;24(1):1492. 39696195 10.1186/s12909-024-06466-z PMC11657493

[33] Fakoya AOJ, Ndrio M, McCarthy KJ. Facilitating active collaborative learning in medical Education; a literature review of peer instruction method. Adv Med Educ Pract. 2023;14:1087–99. 37810958 10.2147/AMEP.S421400 PMC10559896

[34] Dornan T, Littlewood S, Margolis SA, Scherpbier A, Spencer J, Ypinazar V. How can experience in clinical and community settings contribute to early medical education? A BEME systematic review. Med Teach. 2006;28(1):3–18. 16627313 10.1080/01421590500410971

[35] Peduzzi P, Concato J, Kemper E, Holford TR, Feinstein AR. A simulation study of the number of events per variable in logistic regression analysis. J Clin Epidemiol. 1996;49(12):1373–9. 8970487 10.1016/s0895-4356(96)00236-3

[36] Ertl-Wagner B, Barkhausen J, Mahnken AH, Mentzel HJ, Uder M, Weidemann J, Stumpp P. White paper: radiological curriculum for undergraduate medical education in Germany. RoFo: Fortschr Auf Dem Gebiete Der Rontgenstrahlen Und Der Nuklearmedizin. 2016;188(11):1017–23. 10.1055/s-0042-116026 27760438

[37] Konge L, Albrecht-Beste E, Bachmann Nielsen M. Ultrasound in Pre-Graduate Medical Education. *Ultraschall in der Medizin (Stuttgart, Germany: 1980).* 2015;36(3):213–215. 10.1055/s-0034-1399553 26069997

[38] Skadorwa T. Anatomy podcasts for medical education. Clin Anat (New York NY). 2022;35(5):580–91. 10.1002/ca.23865 35363384

[39] Miranda N, Zhang JY, Qiu M. Young glaucoma specialist practice patterns: why do you do what you do? Adv Ophthalmol Pract Res. 2025;5(4):227–34. 41368271 10.1016/j.aopr.2025.07.001 PMC12684888

