# Supplementary material for: Bridging the gap: a survey of resident physicians’ needs for cross-sectional anatomy education and a collaborative teaching framework
Source: BMC Med Educ. 2026 Jan 7;26:197. doi: 10.1186/s12909-026-08567-3 (PMC12870541; doi:10.1186/s12909-026-08567-3)
Supplement: Supplementary file 1 — Supplementary Material 1. [file 12909_2026_8567_MOESM1_ESM.pdf]

Cross-sectional Anatomy Education Needs Assessment Questionnaire for Resident Physicians

Psychometric Properties

Measure	Value
Content Validity Index (CVI)	0.94
Internal Consistency (Cronbach's α)	0.86
Test-retest Reliability (r)	0.87
Pilot Sample for Test-retest	n = 25 (retested after 2 weeks)

Questionnaire Structure

Section	Domain	Items	Questions
A	Demographic Information	5	Q1–Q5
B	Knowledge and Previous Education	5	Q6–Q10
C	Clinical Application and Challenges	7	Q11–Q17
D	Educational Preferences	7	Q18–Q24
	Total	24	

Complete Survey Instrument

Section A: Demographic Information (5 items)

1. What year of residency training are you currently in?

☐ 1st year
☐ 2nd year
☐ 3rd year
☐ 4th year and above

2. Which department/specialty are you affiliated with?

☐ Internal Medicine
☐ Surgery
☐ Other specialties (please specify: _________)

3. What is your age?

☐ 20–25 years
☐ 26–30 years
☐ 31–35 years
☐ 36 years and above

4. What is your gender?

☐ Male
☐ Female

5. What is your highest level of education completed?

☐ Bachelor's degree
☐ Master's degree
☐ Doctoral degree

Section B: Cross-sectional Anatomy Knowledge and Previous Education (5 items)

6. How would you rate your current understanding of cross-sectional anatomy?

☐ Very knowledgeable
☐ Somewhat knowledgeable
☐ Not very knowledgeable
☐ Not knowledgeable at all

7. Have you previously participated in a formal cross-sectional anatomy course?

☐ Yes
☐ No

8. How would you rate your mastery of cross-sectional anatomy teaching content?

(Skip if answered "No" to Q7)

☐ Complete mastery
☐ Good mastery
☐ Average mastery
☐ Poor mastery
☐ No mastery at all

9. How do you think cross-sectional anatomy courses have impacted your clinical work?

(Skip if answered "No" to Q7)

☐ Very significant impact
☐ Some impact
☐ Little impact
☐ No impact

10. In your current studies and clinical work, do you encounter situations requiring cross-sectional anatomy knowledge?

☐ Very frequently (daily)
☐ Frequently (weekly)
☐ Occasionally (monthly)
☐ Rarely
☐ Never

Section C: Clinical Application and Challenges (7 items)

11. How would you rate your current clinical imaging interpretation abilities?

☐ Very proficient
☐ Quite proficient
☐ Average
☐ Not very proficient
☐ Not proficient at all

12. What do you consider your weakest area in clinical imaging interpretation?

☐ Anatomical structure positioning and adjacent relationships
☐ Pathological changes assessment
☐ Lesion localization
☐ Comprehensive diagnosis

13. In the reading process, what do you primarily rely on?

☐ Independent completion
☐ Consulting supervisors/senior physicians
☐ Consulting mentors
☐ Relying on reports

14. Do you feel confident in performing independent imaging interpretation and reading?

☐ Yes, very confident
☐ Sometimes, need help at times
☐ Not very confident, need guidance
☐ No confidence at all, completely dependent on others

15. How effective do you think learning imaging interpretation through clinical experience and other medical courses (such as regional anatomy, systematic anatomy) has been?

☐ Very good
☐ Good
☐ Average
☐ Poor
☐ Very poor

16. In imaging and interpretation, how much help or need do you think cross-sectional anatomy learning provides?

☐ Very high need
☐ High need
☐ Average need
☐ Low need
☐ Very low need

17. What specific clinical content do you think should be included in cross-sectional anatomy courses?

(Multiple selections allowed)

☐ Multi-modal imaging anatomy comparison
☐ Clinical case imaging anatomy analysis
☐ Imaging anatomy-surgical correlation
☐ Other (please specify: _________)

Section D: Educational Preferences (7 items)

18. How important do you think clinical physician participation in cross-sectional anatomy course teaching is?

☐ Very important
☐ Important
☐ Average
☐ Less important

☐ Not important

19. Do you support clinical physicians working together with specialized basic anatomy teachers for teaching?

☐ Strongly support
☐ Support
☐ Average
☐ Do not support
☐ Strongly oppose

20. What advantages do you think clinical physicians working with specialized teachers in collaborative teaching offer?

(Multiple selections allowed)
☐ Providing more clinical cases
☐ Better integration with practical operations
☐ Providing multi-perspective knowledge
☐ Increasing teaching interactivity

21. Which learning format do you prefer?

☐ Classroom lectures
☐ Online courses
☐ Human anatomy museum field study
☐ Self-directed learning

22. If you choose online courses, which type do you prefer?

☐ Recorded lectures
☐ Live streaming courses
☐ Blended teaching (combination of online and offline)

23. What content would you like to see added to the course?

(Multiple selections allowed)
☐ More clinical cases
☐ 3D imaging technology applications
☐ Interactive teaching
☐ Other (please specify: __________)

24. Any additional comments or suggestions regarding cross-sectional anatomy education?

____
____

Survey Information

• Estimated completion time: 15–20 minutes
• All responses are anonymous and voluntary
• Data will be used for educational research purposes only
• Participants may withdraw at any time without repercussions

Thank you for your participation!
